# (*E*)-3-(9-Anthr­yl)-1-(4-chloro­phen­yl)-2-(1*H*-1,2,4-triazol-1-yl)prop-2-en-1-one

**DOI:** 10.1107/S1600536809038628

**Published:** 2009-10-03

**Authors:** Guang-Zhou Wang, Yuan Shi, Kun Wan, Cheng-He Zhou

**Affiliations:** a Laboratory of Bioorganic & Medicinal Chemistry, School of Chemistry and Chemical Engineering, Southwest University, Chongqing 400715, People’s Republic of China

## Abstract

In the title compound, C_25_H_16_ClN_3_O, the anthryl and chloro­phenyl substituents are on opposite sides of the triazole ring. The anthryl and benzene mean planes are aligned at 83.35 (2) and 89.09 (2)°, respectively, with respect to the triazole ring.

## Related literature

For general background to the biological properties of chalcones, see: Corréa *et al.* (2001 ▶). For the synthesis, see: Erhardt *et al.* (1985 ▶); Kranz *et al.* (1980 ▶). For similar crystal structures, see: Lu *et al.* (2009 ▶); Wang *et al.* (2009 ▶); Yan *et al.* (2009 ▶).
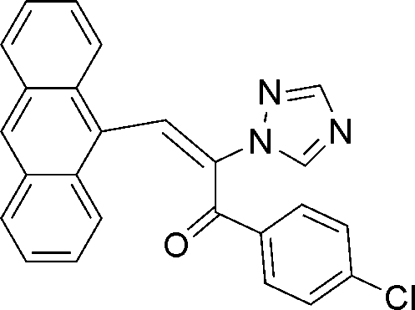


## Experimental

### 

#### Crystal data

C_25_H_16_ClN_3_O
*M* *_r_* = 409.86
Orthorhombic, 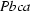

*a* = 13.1464 (11) Å
*b* = 13.5485 (12) Å
*c* = 22.0974 (19) Å
*V* = 3935.9 (6) Å^3^
*Z* = 8
Mo *K*α radiation
μ = 0.22 mm^−1^
*T* = 298 K
0.26 × 0.12 × 0.10 mm

#### Data collection

Bruker SMART diffractometer
Absorption correction: multi-scan (*SADABS*; Sheldrick, 1996 ▶) *T*_min_ = 0.946, *T*_max_ = 0.979
19759 measured reflections
3859 independent reflections
3430 reflections with *I* > 2σ(*I*)
*R*_int_ = 0.119

#### Refinement

*R*[*F* ^2^ > 2σ(*F* ^2^)] = 0.065
*wR*(*F* ^2^) = 0.162
*S* = 1.12
3859 reflections
271 parameters
H-atom parameters constrained
Δρ_max_ = 0.43 e Å^−3^
Δρ_min_ = −0.34 e Å^−3^

### 

Data collection: *SMART* (Bruker, 2001 ▶); cell refinement: *SAINT-Plus* (Bruker, 2001 ▶); data reduction: *SAINT-Plus*; program(s) used to solve structure: *SHELXS97* (Sheldrick, 2008 ▶); program(s) used to refine structure: *SHELXL97* (Sheldrick, 2008 ▶); molecular graphics: *SHELXTL* (Sheldrick, 2008 ▶); software used to prepare material for publication: *SHELXTL*.

## Supplementary Material

Crystal structure: contains datablocks I, global. DOI: 10.1107/S1600536809038628/ng2650sup1.cif

Structure factors: contains datablocks I. DOI: 10.1107/S1600536809038628/ng2650Isup2.hkl

Additional supplementary materials:  crystallographic information; 3D view; checkCIF report

## Figures and Tables

**Fig. 1.**
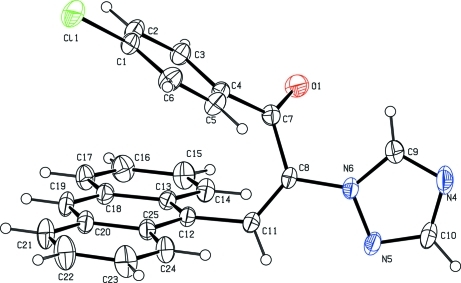
The molecular structure of (I), showing the atom-numbering scheme. Displacement ellipsoids are drawn at the 50% probability level.

**Fig. 2.**
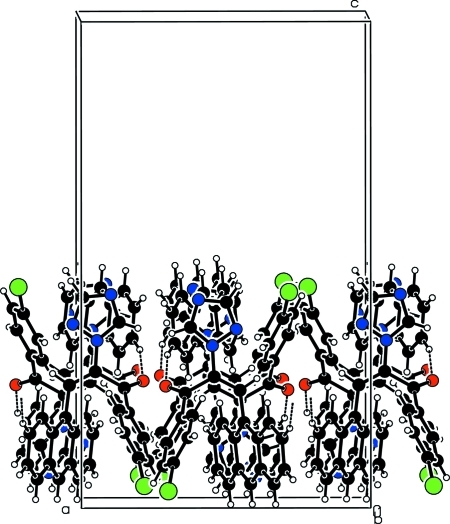
Part of the crystal structure of (I), showing the formation of the three-dimensional network.

## supplementary crystallographic information

### Comment

Chalcone derivatives possess wide biological properties such as antimicrobial, antifungal, antileishmanial, antibacterial, antimalarial, analgesic, anti-inflammatory and chemopreventive activities (Corréa *et al.*, 2001). Recently chalcone-containing derivatives received special attention. Our interest is the research and development of azole-derived chalcones as medicinal agents. We found that all the synthesized imidazole-derived chalcone compounds exhibited significant antimicrobial and anticancer activities, and have reported several crystal structues of nitroimidazole-containing chalcones (Lu *et al.*, 2009; Wang *et al.*, 2009*b*) and a triazole-derived phenyl compound(Yan *et al.*, 2009*c*). In our ongoing research, here we would like to report the crystal structure of the first both triazole and anthracence derived chalcone.

In the crystal structure (Fig. 1), the title compound is non-planar, display a ' *Y *' shape, with the anthryl ring and phenyl moiety on opposite sides of the triazole ring, the anthracene and benzene mean planes make dihedral angles of 83.35 (2) and 89.09 (2)°, respectively, with the plane of the triazole ring. The crystal structure is stabilized by weak intermolecular C—H···O hydrogen bonds.

### Experimental

Compound (I) was synthesized according to the procedure of Erhardt *et al.* (1985); Kranz *et al.*, (1980). A crystal of (I) suitable for X-ray analysis was grown from a mixture solution of chloroform and acetone by slow evaporationat room temperature.

### Refinement

All the hydrogen atoms were placed at their geometrical positions with C—H = 0.93Å and *U*_iso_(H) = 1.2*U*_eq_(C).

### Crystal data

**Table:**

C_25_H_16_ClN_3_O	*F*(000) = 1696
*M**_r_* = 409.86	*D*_x_ = 1.383 Mg m^−^^3^
Orthorhombic, *P**b**c**a*	Mo *K*α radiation, λ = 0.71073 Å
Hall symbol: -P 2ac 2ab	Cell parameters from 6265 reflections
*a* = 13.1464 (11) Å	θ = 2.4–27.5°
*b* = 13.5485 (12) Å	µ = 0.22 mm^−^^1^
*c* = 22.0974 (19) Å	*T* = 298 K
*V* = 3935.9 (6) Å^3^	Block, yellow
*Z* = 8	0.26 × 0.12 × 0.10 mm

### Data collection

**Table:**

Bruker SMART diffractometer	3859 independent reflections
Radiation source: fine-focus sealed tube	3430 reflections with *I* > 2σ(*I*)
graphite	*R*_int_ = 0.119
φ and ω scans	θ_max_ = 26.0°, θ_min_ = 2.4°
Absorption correction: multi-scan (*SADABS*; Sheldrick, 1996)	*h* = −16→16
*T*_min_ = 0.946, *T*_max_ = 0.979	*k* = −16→16
19759 measured reflections	*l* = −17→27

### Refinement

**Table:**

Refinement on *F*^2^	Primary atom site location: structure-invariant direct methods
Least-squares matrix: full	Secondary atom site location: difference Fourier map
*R*[*F*^2^ > 2σ(*F*^2^)] = 0.065	Hydrogen site location: inferred from neighbouring sites
*wR*(*F*^2^) = 0.162	H-atom parameters constrained
*S* = 1.12	*w* = 1/[σ^2^(*F*_o_^2^) + (0.0638*P*)^2^ + 1.7945*P*] where *P* = (*F*_o_^2^ + 2*F*_c_^2^)/3
3859 reflections	(Δ/σ)_max_ < 0.001
271 parameters	Δρ_max_ = 0.43 e Å^−^^3^
0 restraints	Δρ_min_ = −0.34 e Å^−^^3^

### Special details

**Table:**

Geometry. All e.s.d.'s (except the e.s.d. in the dihedral angle between two l.s. planes) are estimated using the full covariance matrix. The cell e.s.d.'s are taken into account individually in the estimation of e.s.d.'s in distances, angles and torsion angles; correlations between e.s.d.'s in cell parameters are only used when they are defined by crystal symmetry. An approximate (isotropic) treatment of cell e.s.d.'s is used for estimating e.s.d.'s involving l.s. planes.
Refinement. Refinement of *F*^2^ against ALL reflections. The weighted *R*-factor *wR* and goodness of fit *S* are based on *F*^2^, conventional *R*-factors *R* are based on *F*, with *F* set to zero for negative *F*^2^. The threshold expression of *F*^2^ > σ(*F*^2^) is used only for calculating *R*-factors(gt) *etc*. and is not relevant to the choice of reflections for refinement. *R*-factors based on *F*^2^ are statistically about twice as large as those based on *F*, and *R*- factors based on ALL data will be even larger.

### Fractional atomic coordinates and isotropic or equivalent isotropic displacement parameters (Å^2^)

**Table:**

	*x*	*y*	*z*	*U*_iso_*/*U*_eq_	
C1	0.30643 (17)	1.1607 (2)	0.39287 (11)	0.0426 (6)	
C2	0.27174 (18)	1.0658 (2)	0.40129 (11)	0.0447 (6)	
H2	0.2393	1.0481	0.4371	0.054*	
C3	0.28585 (17)	0.99743 (18)	0.35589 (11)	0.0391 (5)	
H3	0.2607	0.9337	0.3604	0.047*	
C4	0.33774 (15)	1.02351 (17)	0.30331 (9)	0.0330 (5)	
C5	0.37216 (18)	1.11961 (18)	0.29626 (11)	0.0421 (6)	
H5	0.4068	1.1373	0.2612	0.051*	
C6	0.35564 (19)	1.18896 (19)	0.34055 (12)	0.0474 (6)	
H6	0.3772	1.2538	0.3353	0.057*	
C7	0.35073 (16)	0.95057 (16)	0.25405 (10)	0.0346 (5)	
C8	0.45084 (15)	0.95049 (15)	0.22047 (9)	0.0303 (5)	
C9	0.3687 (2)	0.9653 (2)	0.11838 (12)	0.0535 (7)	
H9	0.3044	0.9863	0.1304	0.064*	
C10	0.4936 (2)	0.9244 (2)	0.06792 (11)	0.0484 (6)	
H10	0.5345	0.9105	0.0347	0.058*	
C11	0.54031 (16)	0.94857 (17)	0.24822 (9)	0.0340 (5)	
H11	0.5985	0.9497	0.2243	0.041*	
C12	0.55381 (15)	0.94475 (17)	0.31479 (9)	0.0327 (5)	
C13	0.52315 (15)	0.86077 (17)	0.34745 (9)	0.0332 (5)	
C14	0.48195 (18)	0.77419 (18)	0.32006 (11)	0.0406 (5)	
H14	0.4733	0.7724	0.2783	0.049*	
C15	0.4552 (2)	0.6945 (2)	0.35349 (13)	0.0506 (6)	
H15	0.4292	0.6389	0.3343	0.061*	
C16	0.4662 (2)	0.6944 (2)	0.41698 (12)	0.0517 (7)	
H16	0.4470	0.6393	0.4394	0.062*	
C17	0.5045 (2)	0.7742 (2)	0.44502 (11)	0.0464 (6)	
H17	0.5110	0.7735	0.4869	0.056*	
C18	0.53536 (16)	0.85998 (18)	0.41238 (10)	0.0376 (5)	
C19	0.57685 (18)	0.94139 (19)	0.44096 (10)	0.0423 (6)	
H19	0.5826	0.9411	0.4829	0.051*	
C20	0.61025 (17)	1.02352 (18)	0.40905 (10)	0.0386 (5)	
C21	0.6548 (2)	1.1069 (2)	0.43795 (12)	0.0534 (7)	
H21	0.6603	1.1075	0.4799	0.064*	
C22	0.6890 (3)	1.1848 (2)	0.40624 (14)	0.0653 (8)	
H22	0.7177	1.2383	0.4263	0.078*	
C23	0.6813 (2)	1.1853 (2)	0.34226 (13)	0.0590 (7)	
H23	0.7065	1.2386	0.3204	0.071*	
C24	0.63764 (19)	1.10861 (19)	0.31269 (11)	0.0450 (6)	
H24	0.6321	1.1108	0.2708	0.054*	
C25	0.60008 (16)	1.02502 (17)	0.34426 (9)	0.0345 (5)	
Cl1	0.28705 (6)	1.24841 (6)	0.44918 (4)	0.0647 (3)	
N4	0.39691 (19)	0.95338 (19)	0.06181 (9)	0.0571 (6)	
N5	0.52736 (15)	0.91671 (17)	0.12348 (9)	0.0449 (5)	
N6	0.44473 (13)	0.94308 (14)	0.15650 (8)	0.0334 (4)	
O1	0.28455 (12)	0.89224 (14)	0.24077 (9)	0.0505 (5)	

### Atomic displacement parameters (Å^2^)

**Table:**

	*U*^11^	*U*^22^	*U*^33^	*U*^12^	*U*^13^	*U*^23^
C1	0.0445 (12)	0.0499 (15)	0.0335 (12)	0.0064 (11)	0.0001 (9)	−0.0160 (11)
C2	0.0506 (14)	0.0532 (16)	0.0303 (12)	0.0020 (11)	0.0086 (10)	−0.0021 (11)
C3	0.0449 (12)	0.0371 (13)	0.0354 (12)	−0.0013 (10)	0.0042 (9)	0.0017 (10)
C4	0.0348 (11)	0.0350 (12)	0.0293 (11)	0.0028 (9)	0.0000 (8)	−0.0028 (9)
C5	0.0513 (13)	0.0387 (13)	0.0364 (13)	−0.0036 (10)	0.0105 (10)	−0.0026 (10)
C6	0.0573 (15)	0.0347 (13)	0.0503 (15)	−0.0030 (11)	0.0081 (11)	−0.0093 (11)
C7	0.0378 (11)	0.0346 (12)	0.0313 (11)	0.0009 (9)	−0.0031 (8)	−0.0021 (9)
C8	0.0418 (11)	0.0280 (11)	0.0211 (10)	0.0010 (8)	0.0003 (8)	−0.0030 (8)
C9	0.0489 (14)	0.078 (2)	0.0332 (13)	0.0122 (13)	−0.0078 (10)	−0.0027 (13)
C10	0.0687 (16)	0.0541 (16)	0.0225 (11)	0.0060 (13)	0.0009 (11)	−0.0051 (11)
C11	0.0397 (11)	0.0380 (12)	0.0244 (11)	0.0011 (9)	0.0025 (8)	−0.0015 (9)
C12	0.0365 (11)	0.0386 (12)	0.0230 (11)	0.0049 (9)	−0.0012 (8)	−0.0014 (9)
C13	0.0358 (11)	0.0377 (12)	0.0260 (11)	0.0070 (9)	0.0000 (8)	−0.0019 (9)
C14	0.0520 (13)	0.0389 (13)	0.0309 (12)	0.0042 (10)	−0.0024 (10)	−0.0025 (10)
C15	0.0654 (16)	0.0412 (15)	0.0454 (15)	−0.0044 (12)	−0.0060 (12)	0.0003 (12)
C16	0.0632 (16)	0.0476 (16)	0.0444 (15)	−0.0039 (12)	−0.0039 (12)	0.0151 (12)
C17	0.0566 (15)	0.0524 (16)	0.0303 (12)	0.0013 (12)	−0.0055 (10)	0.0108 (11)
C18	0.0423 (12)	0.0432 (13)	0.0272 (11)	0.0060 (10)	−0.0009 (9)	0.0012 (10)
C19	0.0568 (14)	0.0494 (15)	0.0208 (11)	0.0048 (11)	−0.0032 (9)	−0.0036 (10)
C20	0.0487 (13)	0.0406 (13)	0.0264 (11)	0.0074 (10)	−0.0045 (9)	−0.0051 (10)
C21	0.0813 (19)	0.0464 (15)	0.0324 (13)	−0.0027 (13)	−0.0121 (12)	−0.0104 (11)
C22	0.102 (2)	0.0429 (16)	0.0509 (17)	−0.0114 (15)	−0.0188 (16)	−0.0087 (13)
C23	0.087 (2)	0.0419 (15)	0.0486 (16)	−0.0120 (14)	−0.0113 (14)	0.0063 (12)
C24	0.0604 (15)	0.0420 (14)	0.0325 (12)	−0.0027 (11)	−0.0076 (10)	0.0040 (10)
C25	0.0397 (11)	0.0376 (12)	0.0261 (11)	0.0057 (9)	−0.0041 (8)	−0.0021 (9)
Cl1	0.0712 (5)	0.0703 (5)	0.0526 (5)	0.0044 (4)	0.0062 (3)	−0.0339 (4)
N4	0.0731 (15)	0.0724 (17)	0.0258 (11)	0.0103 (13)	−0.0114 (10)	−0.0013 (10)
N5	0.0533 (11)	0.0574 (13)	0.0240 (10)	0.0118 (10)	0.0013 (8)	−0.0069 (9)
N6	0.0422 (10)	0.0347 (10)	0.0234 (9)	0.0036 (8)	−0.0032 (7)	−0.0042 (7)
O1	0.0446 (9)	0.0519 (11)	0.0550 (11)	−0.0086 (8)	0.0035 (8)	−0.0199 (9)

### Geometric parameters (Å, °)

**Table:**

C1—C2	1.377 (4)	C12—C13	1.406 (3)
C1—C6	1.379 (4)	C13—C14	1.427 (3)
C1—Cl1	1.740 (2)	C13—C18	1.444 (3)
C2—C3	1.378 (3)	C14—C15	1.355 (4)
C2—H2	0.9300	C14—H14	0.9300
C3—C4	1.393 (3)	C15—C16	1.411 (4)
C3—H3	0.9300	C15—H15	0.9300
C4—C5	1.387 (3)	C16—C17	1.344 (4)
C4—C7	1.480 (3)	C16—H16	0.9300
C5—C6	1.374 (3)	C17—C18	1.427 (4)
C5—H5	0.9300	C17—H17	0.9300
C6—H6	0.9300	C18—C19	1.383 (3)
C7—O1	1.211 (3)	C19—C20	1.388 (4)
C7—C8	1.511 (3)	C19—H19	0.9300
C8—C11	1.327 (3)	C20—C21	1.424 (3)
C8—N6	1.419 (3)	C20—C25	1.438 (3)
C9—N4	1.314 (3)	C21—C22	1.344 (4)
C9—N6	1.342 (3)	C21—H21	0.9300
C9—H9	0.9300	C22—C23	1.417 (4)
C10—N5	1.310 (3)	C22—H22	0.9300
C10—N4	1.337 (4)	C23—C24	1.355 (4)
C10—H10	0.9300	C23—H23	0.9300
C11—C12	1.483 (3)	C24—C25	1.419 (3)
C11—H11	0.9300	C24—H24	0.9300
C12—C25	1.406 (3)	N5—N6	1.356 (3)
			
C2—C1—C6	121.9 (2)	C15—C14—H14	119.2
C2—C1—Cl1	119.53 (19)	C13—C14—H14	119.2
C6—C1—Cl1	118.6 (2)	C14—C15—C16	121.1 (2)
C1—C2—C3	119.0 (2)	C14—C15—H15	119.5
C1—C2—H2	120.5	C16—C15—H15	119.5
C3—C2—H2	120.5	C17—C16—C15	119.8 (2)
C2—C3—C4	120.2 (2)	C17—C16—H16	120.1
C2—C3—H3	119.9	C15—C16—H16	120.1
C4—C3—H3	119.9	C16—C17—C18	121.9 (2)
C5—C4—C3	119.4 (2)	C16—C17—H17	119.0
C5—C4—C7	120.4 (2)	C18—C17—H17	119.0
C3—C4—C7	120.0 (2)	C19—C18—C17	122.1 (2)
C6—C5—C4	120.7 (2)	C19—C18—C13	119.5 (2)
C6—C5—H5	119.7	C17—C18—C13	118.5 (2)
C4—C5—H5	119.7	C18—C19—C20	122.1 (2)
C5—C6—C1	118.8 (2)	C18—C19—H19	118.9
C5—C6—H6	120.6	C20—C19—H19	118.9
C1—C6—H6	120.6	C19—C20—C21	122.6 (2)
O1—C7—C4	122.1 (2)	C19—C20—C25	119.2 (2)
O1—C7—C8	120.4 (2)	C21—C20—C25	118.3 (2)
C4—C7—C8	117.53 (18)	C22—C21—C20	121.8 (2)
C11—C8—N6	120.60 (18)	C22—C21—H21	119.1
C11—C8—C7	123.04 (19)	C20—C21—H21	119.1
N6—C8—C7	116.10 (17)	C21—C22—C23	120.0 (3)
N4—C9—N6	111.1 (2)	C21—C22—H22	120.0
N4—C9—H9	124.5	C23—C22—H22	120.0
N6—C9—H9	124.5	C24—C23—C22	120.5 (3)
N5—C10—N4	116.1 (2)	C24—C23—H23	119.8
N5—C10—H10	121.9	C22—C23—H23	119.8
N4—C10—H10	121.9	C23—C24—C25	121.5 (2)
C8—C11—C12	124.43 (19)	C23—C24—H24	119.3
C8—C11—H11	117.8	C25—C24—H24	119.3
C12—C11—H11	117.8	C12—C25—C24	122.7 (2)
C25—C12—C13	120.81 (19)	C12—C25—C20	119.4 (2)
C25—C12—C11	119.0 (2)	C24—C25—C20	117.9 (2)
C13—C12—C11	120.2 (2)	C9—N4—C10	102.0 (2)
C12—C13—C14	123.8 (2)	C10—N5—N6	102.22 (19)
C12—C13—C18	118.9 (2)	C9—N6—N5	108.56 (19)
C14—C13—C18	117.2 (2)	C9—N6—C8	130.66 (19)
C15—C14—C13	121.5 (2)	N5—N6—C8	120.59 (17)
			
C6—C1—C2—C3	−0.6 (4)	C14—C13—C18—C19	178.7 (2)
Cl1—C1—C2—C3	178.79 (19)	C12—C13—C18—C17	−179.7 (2)
C1—C2—C3—C4	2.3 (4)	C14—C13—C18—C17	−0.9 (3)
C2—C3—C4—C5	−2.0 (3)	C17—C18—C19—C20	177.7 (2)
C2—C3—C4—C7	−178.6 (2)	C13—C18—C19—C20	−1.9 (3)
C3—C4—C5—C6	0.1 (4)	C18—C19—C20—C21	−178.8 (2)
C7—C4—C5—C6	176.6 (2)	C18—C19—C20—C25	1.0 (3)
C4—C5—C6—C1	1.6 (4)	C19—C20—C21—C22	178.3 (3)
C2—C1—C6—C5	−1.4 (4)	C25—C20—C21—C22	−1.5 (4)
Cl1—C1—C6—C5	179.2 (2)	C20—C21—C22—C23	−0.1 (5)
C5—C4—C7—O1	−138.9 (2)	C21—C22—C23—C24	1.5 (5)
C3—C4—C7—O1	37.7 (3)	C22—C23—C24—C25	−1.2 (5)
C5—C4—C7—C8	41.5 (3)	C13—C12—C25—C24	176.2 (2)
C3—C4—C7—C8	−142.0 (2)	C11—C12—C25—C24	−2.1 (3)
O1—C7—C8—C11	−129.5 (2)	C13—C12—C25—C20	−3.9 (3)
C4—C7—C8—C11	50.1 (3)	C11—C12—C25—C20	177.82 (19)
O1—C7—C8—N6	44.7 (3)	C23—C24—C25—C12	179.6 (2)
C4—C7—C8—N6	−135.7 (2)	C23—C24—C25—C20	−0.4 (4)
N6—C8—C11—C12	−173.0 (2)	C19—C20—C25—C12	1.9 (3)
C7—C8—C11—C12	1.0 (4)	C21—C20—C25—C12	−178.2 (2)
C8—C11—C12—C25	−115.3 (3)	C19—C20—C25—C24	−178.1 (2)
C8—C11—C12—C13	66.4 (3)	C21—C20—C25—C24	1.7 (3)
C25—C12—C13—C14	−175.7 (2)	N6—C9—N4—C10	−0.8 (3)
C11—C12—C13—C14	2.5 (3)	N5—C10—N4—C9	0.4 (4)
C25—C12—C13—C18	3.0 (3)	N4—C10—N5—N6	0.1 (3)
C11—C12—C13—C18	−178.76 (19)	N4—C9—N6—N5	0.9 (3)
C12—C13—C14—C15	178.8 (2)	N4—C9—N6—C8	175.9 (2)
C18—C13—C14—C15	0.1 (3)	C10—N5—N6—C9	−0.6 (3)
C13—C14—C15—C16	0.6 (4)	C10—N5—N6—C8	−176.1 (2)
C14—C15—C16—C17	−0.5 (4)	C11—C8—N6—C9	−162.8 (3)
C15—C16—C17—C18	−0.4 (4)	C7—C8—N6—C9	22.9 (4)
C16—C17—C18—C19	−178.6 (2)	C11—C8—N6—N5	11.6 (3)
C16—C17—C18—C13	1.1 (4)	C7—C8—N6—N5	−162.7 (2)
C12—C13—C18—C19	−0.1 (3)		

### Hydrogen-bond geometry (Å, °)

**Table:**

*D*—H···*A*	*D*—H	H···*A*	*D*···*A*	*D*—H···*A*
C23—H23···O1^i^	0.93	2.48	3.381 (3)	162

Symmetry codes: (i) −*x*+1, *y*+1/2, −*z*+1/2.
